# 

**Published:** 1884-01

**Authors:** 


					﻿Vol. V.
JANUARY, 1884.
No. 1.
THE
man twn pr it liionre
> Monfthly Journal
DEVOTED TO
DENTAL AND ORAL SCIENCE
W. C. BARRETT, M. D., D. D. S.,
EDITOR.
The Hew York Dental Journal Association,
PUBLISHERS,
N"o. 35 West LCStli Street, New Yorlc.
$2.50 Per Annum in Advance, .... Single Copies, 25 Cents.
Entered at the Post-Office, Buffalo, N. V., as Second-Class matter.
				

